# Is Recurving an Effective Strategy of *Trifolium repens* L. to Augment Reproduction?

**DOI:** 10.1155/2016/4741086

**Published:** 2016-02-29

**Authors:** Mustaqeem Ahmad, Sanjay Kr. Uniyal

**Affiliations:** Adaptation Biology and Climate Change Division, CSIR-Institute of Himalayan Bioresource Technology, Palampur, Himachal Pradesh 176061, India

## Abstract

Flowers of* Trifolium repens* L. show recurving. We, therefore, studied whether this is an effective strategy employed by the species to augment reproduction. For this, fifty plants of the species were tagged and monitored. This included twenty control and twenty constrained to recurve. The remaining 10 plants were covered with a net to limit cross-pollination. Daily observations on the plants were recorded. No significant difference in the number of flowers per inflorescence between control and constrained plants was found. However, a significant difference (*p* < 0.001) in the number of seeds produced by control (68.35 ± 3.92) and constrained plants (22.25 ± 1.35) was observed. Three times more seeds, without compromising on seed mass (*p* > 0.05), were produced in the control plants. No seeds were produced in the flowers that were netted. Thus, recurving appears to help* T. repens* in reproduction.

## 1. Introduction

**Figure 1:**
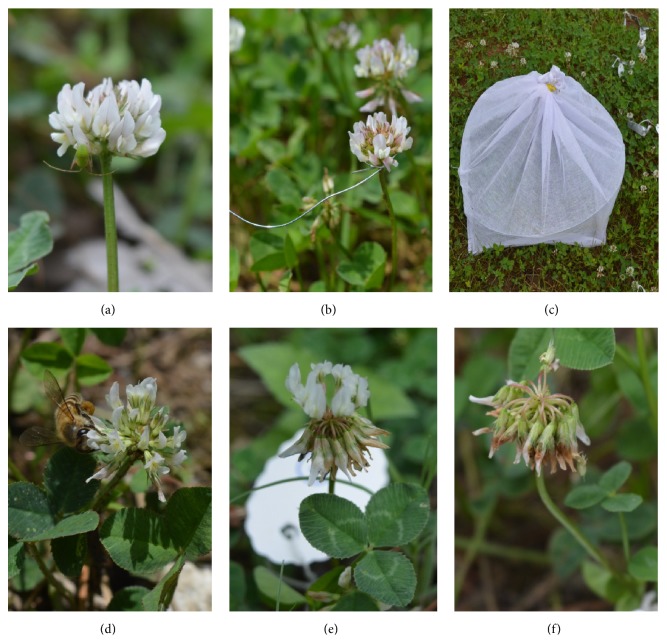
Inflorescence of* Trifolium repens* ((a) compact inflorescence with flower pointing upwards, (b) a constrained flower, (c) plants covered with net to deter pollination, (d) a pollinator on the peripheral mature flower, (e) half of the flowers have recurved while the ones yet to be fertilized face upwards, and (f) inflorescence completely recurved with fruit formation in the process).

Recurving refers to bending of a structure or an organ in the direction opposite to that of its growth [1]. The inflorescence of* Trifolium repens* L. presents an excellent example of recurving (Figure 1). The species commonly referred to as “white clover” is a small herbaceous plant of the family Fabaceae. The taxa owes its name to the three leaflets that are characteristic of the species [2].

The species is widely distributed across the globe and in the Himalaya it prominently occurs between 1000 and 2500 m asl [3]. This wide distribution, in addition to its adaptive amplitude, calls for the species to have an effective sexual reproductive strategy. This is because while vegetative propagation may allow for small scale colonization of the species, sexual reproduction ensures genetic variability and long range dispersal [4, 5].

Being largely an insect pollinated species, attracting insects is key to the success of* T. repens* [6]. No doubt then that inflorescence is the most visible and prominent part of the plant. We found that in a mature plant more than 30% of the above-ground phytomass is accounted for by the inflorescence (excluding the peduncle). The inflorescence is a globular raceme (15–25 mm in width) that is supported by a large peduncle. The flowers are white with a tinge of pink [7]. The number of flowers in an inflorescence ranges between 20 and 150 and each flower measures 8–10 mm in length [8]. Once mature and fertilized, these individual flowers progressively recurve such that on a temporal scale the entire inflorescence takes a 180-degree turn (Figure 1). We, therefore, studied whether recurving is an effective strategy employed by* T. repens* to maximize chances of fertilization and thereby seed production.

## 2. Methods

Fifty individuals of* T. repens* were identified and tagged in wild during the month of June 2015. Of these, twenty were control (growing as such) and twenty were constrained from recurving. The remaining ten plants were covered with net to limit cross-pollination. In order to constrain flowers, a wire loop was placed below the inflorescence such that it constrained recurving but did not damage the inflorescence (Figure 1). All these plants occupied the same habitat and grew in the same natural environment.

The study site lies at coordinates 32° 06′ 12. 82′′ N and 76° 33′ 25.55′′ E at an altitude of 1300 m in the western Himalayan state of Himachal Pradesh. Subtropical chir-pine vegetation dominates the area. Annual rainfall at the site is ~2400 mm, maximum of which occurs during the month of August (~845 mm). On the other hand, January is the coldest month with a mean temperature of 8.48 °C while June is the warmest month that reports a mean temperature of 27.28 °C.

The plants were of similar age, as we had been monitoring them for growth, and thus it is assumed that they did not differ in fecundity. Daily visit, in the morning, was made to the site and recording of flower characteristics was done. We noted the flower colour using the RHRS colour chart [9]. The number of flowers per inflorescence was noted. Frequency of flower recurving, days to total flower recurving, and number of seeds per fruit were recorded. Seeds were later collected and weighed on a digital scale to get an idea of seed mass.

The data so collected was analyzed with respect to number of flower in inflorescence, total days to recurving, number of seeds, and seed mass per inflorescence. The same has been compared between the control and constrained plants. Descriptive statistics has been worked out and comparisons have been done using *t*-test in Ri386.3.1.3 environment [10].

## 3. Results and Discussion

Flowers of the species belonged to white colour group 155A that on maturity changed to grey white (156A) with a tinge of pink. These group numbers refer to the colour codes provided by The Royal Horticulture Society, London [9], such that uniformity of colour reporting is maintained.

The number of flowers per inflorescence ranged between 25 and 63 and their mean in control and constrained plants was 44.05 ± 1.71 and 43.80 ± 1.94, respectively (Table 1). The number of flowers per inflorescence did not vary significantly between the control and constrained plants (*t*-test, *p* > 0.05).

Flower size strongly influences pollination as pollinators respond to floral display [1].* Trifolium repens*, possibly, attracts insects by having compactly placed numerous flowers as a single entity. In close cohesive group, its visibility increases. However, while the inflorescence may help in attracting insects, mature flowers are exposed for fertilization which progressively recurve and expose other flowers for fertilization.

A significant difference in duration to recurving or discolouration of flowers was recorded for control and constrained plants (*t*-test, *p* < 0.001). While for the former it was 7.20 ± 0.55 days (recurving), for the later it was 11.20 ± 0.64 days (discolouration) (Table 1). As evident, this phenophase completed at an early date in control plants. As the species mostly occurs in disturbed habitats and has a short growing period, this provides plants with a temporal advantage wherein they can complete their life cycle relatively fast. This may also provide the species with additional time for raising a new progeny and chances of multiple regeneration. It was observed that the flowers on the periphery of the inflorescence mature first and then progressively the inner ones mature.

Once the outer mature flowers have been fertilized, they recurve (Figure 1). By this time the next layer of inner flowers attains maturity and is ready for fertilization. Recurving of the outer flowers provides the much needed space to the inner unfertilized flowers to get exposed and come in contact with the pollinators (Figure 1). Honeybee is considered to be the main pollinator of white clover [11]. Recurving also provides a clue so that searching efforts of the pollinator are minimized. Once these flowers have also been fertilized, they follow the same path till the innermost flowers are fertilized (Figure 1).

Flower orientation and colour changes have an important role in pollination as has also been documented by other workers [12, 13]. In* Geranium refractum*, it has been reported that the downward-facing floral orientation enhances pollen transfer [14]. Similar patterns of changes in floral orientation in* Corydalis sheareri* have been reported to affect pollination efficiency [15]. Interestingly,* Desmodium setigerum* shows a unique ability of reversing its flower colour and shape in case they are not effectively pollinated [16].

Successful pollination and fertilization lead to formation of pods and each pod, in both control and constrained plants, was found to harbour three to four seeds. No seeds were produced in the netted plants. The total seed numbers per inflorescence varied significantly (*t*-test, *p* < 0.001) between the control and constrained plants. Three times more seeds were produced in control plants (68.35 ± 3.92) as compared to the ones that were constrained (22.25 ± 1.35). At the same time, seed mass did not show any significant difference (*t*-test, *p* > 0.05) between the control (0.00048 ± 0.000041 gm) and constrained plants (0.00039 ± 0.000037 gm). Thus, showing that increase in seed number did not compromise seed mass (Table 1). Needless to say, the higher the propagule load, the higher the chances of germination, growth, and dispersal. As reported by others [7, 17], the seeds are heart shaped, smooth, and yellowish brown in colour. Multiple modes of dispersal have been reported for the species. Long distance dispersal of the seeds is ensured through human activities, birds, and grazing animals [6]. Thus, it appears that recurving is an effective strategy employed by the species to augment reproduction.

## 4. Conclusions

The study reveals that recurving of the flowers is an important reproductive strategy of the plant. Owing to this, the species is able maximize fertilization and seed set.

## Figures and Tables

**Table 1:** Comparative account of the recorded parameters between the control and constrained plants (*n* = 20, each).

Parameters	Control	Constrained	*p* value
Number of flowers/inflorescence	44.05 ± 1.71	43.80 ± 1.94	>0.05
Number of seeds/inflorescence	68.35 ± 3.92	22.25 ± 1.35	<0.001
Time to recurving/discolouration (days)	7.20 ± 0.55	11.20 ± 0.64	<0.001
Seed mass (gm)	0.00048 ± 0.000041	0.00039 ± 0.000037	>0.05
